# Cerebral Autoregulation Assessment Using the Near Infrared Spectroscopy ‘NIRS-Only’ High Frequency Methodology in Critically Ill Patients: A Prospective Cross-Sectional Study

**DOI:** 10.3390/cells11142254

**Published:** 2022-07-21

**Authors:** Jeanette Tas, Nick Eleveld, Melisa Borg, Kirsten D. J. Bos, Anne P. Langermans, Sander M. J. van Kuijk, Iwan C. C. van der Horst, Jan Willem J. Elting, Marcel J. H. Aries

**Affiliations:** 1 Department of Intensive Care Medicine, Maastricht University Medical Center+, University Maastricht, 6229 HX Maastricht, The Netherlands; melisa.borg@gmx.de (M.B.); kirstenbos10@gmail.com (K.D.J.B.); annelangermans98@gmail.com (A.P.L.); iwan.vander.horst@mumc.nl (I.C.C.v.d.H.); marcel.aries@mumc.nl (M.J.H.A.); 2 School for Mental Health and Neuroscience (MHeNS), University Maastricht, 6229 HX Maastricht, The Netherlands; 3 Department of Neurology and Clinical Neurophysiology, University Medical Center Groningen, 9713 GZ Groningen, The Netherlands; n.eleveld@umcg.nl (N.E.); j.w.j.elting@umcg.nl (J.W.J.E.); 4 Department of Clinical Epidemiology and Medical Technology Assessment, (KEMTA), Maastricht University Medical Center+, 6229 HX Maastricht, The Netherlands; sander.van.kuijk@mumc.nl; 5 Cardiovascular Research Institute Maastricht (CARIM), 6229 HX Maastricht, The Netherlands

**Keywords:** cerebral autoregulation, near infrared spectroscopy, capillary transit time, oxyhemoglobin, deoxyhemoglobin, transfer function analysis

## Abstract

Impairments in cerebral autoregulation (CA) are related to poor clinical outcome. Near infrared spectroscopy (NIRS) is a non-invasive technique applied to estimate CA. Our general purpose was to study the clinical feasibility of a previously published ‘NIRS-only’ CA methodology in a critically ill intensive care unit (ICU) population and determine its relationship with clinical outcome. Bilateral NIRS measurements were performed for 1–2 h. Data segments of ten-minutes were used to calculate transfer function analyses (TFA) CA estimates between high frequency oxyhemoglobin (oxyHb) and deoxyhemoglobin (deoxyHb) signals. The phase shift was corrected for serial time shifts. Criteria were defined to select TFA phase plot segments (segments) with ‘high-pass filter’ characteristics. In 54 patients, 490 out of 729 segments were automatically selected (67%). In 34 primary neurology patients the median (q1–q3) low frequency (LF) phase shift was higher in 19 survivors compared to 15 non-survivors (13° (6.3–35) versus 0.83° (−2.8–13), *p* = 0.0167). CA estimation using the NIRS-only methodology seems feasible in an ICU population using segment selection for more robust and consistent CA estimations. The ‘NIRS-only’ methodology needs further validation, but has the advantage of being non-invasive without the need for arterial blood pressure monitoring.

## 1. Introduction

Impaired cerebral autoregulation (CA) contributes to a poor clinical outcome in several acute neurological insults (events) such as traumatic brain injury [1], out of hospital cardiac arrest [2] and to the development of delayed cerebral ischemia in subarachnoid hemorrhage [3]. CA impairment has been observed in other critically ill patients like those with sepsis or septic shock and has been shown to be associated with sepsis-associated delirium [4]. However, it is unknown whether this is an indication of systemic hemodynamic failure, (focal) end-organ cerebral damage or more a combination of both. Excursions of arterial blood pressure (ABP) below the lower limit of CA and not absolute ABP were independently associated with postoperative acute kidney injury in cardiac surgery patients [5]. CA monitoring might be a novel method for precise guiding of ABP targets in critically ill patients with a diversity of clinical diagnoses [6].

Near infrared spectroscopy (NIRS) is a non-invasive technique to study cerebral hemodynamics and CA status. In the time domain, the correlation between slow waves in ABP and NIRS-based regional oxygen saturation (rSO_2_) can be calculated as CA trend measures [7]. In the frequency domain, transfer function analysis (TFA) is a common CA methodology which is historically applied using transcranial Doppler (TCD) and ABP recordings over shorter time periods compared to time domain analysis. However, there are only limited reports on TFA using low frequency sampling NIRS devices as used for time domain analysis [8]. In 2018, Elting et al. developed a ‘NIRS-only’ methodology that allows CA estimations by studying the relationship between 50 Hz oxyhemoglobin (oxyHb) and deoxyhemoglobin (deoxyHb) concentration differences in the very low (VLF) and low frequency (LF) range [9]. The phase shifts in these CA frequency ranges were corrected for the capillary transit time (TT blood flow) and the cerebral blood flow/volume ratio (%BF), using the high frequency (HF) range. The correction was applied to correct for the non-CA related ‘group delay’ and ‘washout’ phenomenon. Both at rest and during hypocapnia/hypercapnia, the corrected NIRS phase shifts showed comparable changes in CA status to those measured with TCD and ABP in healthy subjects. In contrast to TCD, NIRS is easy to use, user independent and therefore suitable for a larger population who might benefit from cerebral monitoring. In addition, the ‘NIRS-only’ methodology does not require continuous (invasive or non-invasive) ABP recordings.

An important requirement for reliable CA estimation is that sufficient slow ABP oscillations are present during the recording period [10]. In comatose patients, spontaneous slow oscillations may be limited due to sedation, analgesia and hemodynamic management [11]. The Cerebrovascular Research Network (CARNet) recommends a minimum duration of five minutes for reliable CA estimations with TCD recordings [8]. Zhang et al. showed that after seven minutes of spontaneous ABP oscillations, the TCD-based CA-parameters became stable in an intensive care unit (ICU) population [12].

In this prospective study, the general purpose was to study the clinical feasibility of the ‘NIRS-only’ CA methodology in a critically ill ICU population. The feasibility aims included: (1) developing an automated data processing method. We defined criteria to automatically select data segments for interpretable LF-phase shift estimation and (2) evaluating the clinical applicability by calculating the variability of the LF-phase shift between and within patients. Finally, we studied the relationship between LF-phase shift and the six-month clinical outcome.

## 2. Materials and Methods

Details about the methodology are described in Supplementary File S1. This was a single center, prospective cross-sectional study that was conducted between June 2018 and March 2020. Recruited patients were admitted to the ICU of an academic teaching hospital. The measurement protocol was approved by the local ethical committee (METC Maastricht 16-4-243). The study is reported according to the Strengthening the Reporting of Observational Studies in Epidemiology (STROBE) reporting guidelines [13] (Supplementary Table S1).

### 2.1. Participants

Inclusion criteria were (1) critically ill adult patient (≥18 years and no intention to withdraw treatment), (2) comatose or sedated (Richmond agitation and sedation scale of −4 or −5), (3) intubated and ventilated patient and (4) the ability to measure within 48 h after ICU admission. Excluded patients had (uni- or bilateral) frontotemporal skin hematoma (due to difficulties with obtaining NIRS signals), frequent cardiac arrythmias (mainly atrial fibrillation) or no written informed consent by a lawful representative. The informed consent procedure was in accordance with the declaration of Helsinki’s ethical guidelines [14]. Patients were recruited during daytime when a member of the research team was available. We did not perform a sample size calculation beforehand as there was no literature on ‘NIRS-only’ CA-assessment in critically ill patients at that time.

### 2.2. Near infrared spectroscopy (NIRS) Measurements

NIRS measurements (Portalite, Artinis Medical Systems, Elst, The Netherlands) were performed bilaterally on the frontotemporal regions simultaneously (intended total duration measurement: 1 h) in the period June 2018 until June 2019. This was changed to unilateral, consecutive measurements (intended duration total measurement: 2 h) onwards due to severe issues with crosstalk between the simultaneously acquired NIRS signals. Changes in oxyHb and deoxyHb concentrations were computed using the modified Lambert-Beer law and updated at 50 Hz. During the measurement, nursing interventions (like turning and suctioning) as well as major changes in medication and ventilator settings were limited to a minimum, if the clinical situation allowed.

### 2.3. Data Collection

For each patient, we collected the patient and admission characteristics. The six-month Glasgow Outcome Scale Extended (GOSE) was collected as clinical outcome parameter by telephone interview. In addition, we collected continuous (50 Hz) physiological data: ABP (mmHg), heart rate (HR, min^−1^), electrocardiogram (ECG, μV), end tidal carbon dioxide (EtCO_2_, kPa), peripheral oxygen saturation (SpO_2_, %) and body temperature (°C). This was collected from the Intellivue Philips bedside monitor (Philips MX800, Eindhoven, The Netherlands). OxyHb (μM) and deoxyHb (μM) concentration differences were collected with Oxysoft at 50 Hz recording software (version: 3.0.103.3, Artinis Medical Systems, Elst, The Netherlands). All the (neuro) physiological signals were stored in the research software ICM+^®^ (Cambridge Enterprise, Cambridge, UK).

### 2.4. Data Preparation

All stored data were exported from ICM+ software to plain text files and imported in MATLAB (Release 2019b, The MathWorks, Inc., Natick, MA, USA). NIRS data was first visually inspected, and artifacts were removed (details on the applied methodology can be found in Supplementary Figure S1). Then, the data was stored in ten-minute data segments per patient and imported into a custom made LabVIEW program (LabVIEW 2015, National Instruments, Austin, TX, USA) calculating the CA-estimates (Section 2.4.1) as used in Elting et al. [9]. Sufficiently slow ABP oscillations are required for a reliable CA assessment [10]. The amount of slow ABP and resulting slow oxyHb and deoxyHb oscillations were quantified by the Power Spectral Density (PSD).

#### 2.4.1. Transfer Function Analysis (TFA)

TFA was applied in concordance with the CARNet recommendations for TFA [8]. In addition, Box 1 describes the ‘NIRS-only’ methodology for phase shift correction. The following outputs were obtained for each frequency range of interest: very low frequency (VLF 0.02–0.07 Hz), low frequency (LF 0.07–0.2 Hz) and high frequency range (HF 0.2–0.5 Hz): coherence, gain, corrected phase shift (referred to as ‘phase shift’ in the remaining text) and uncorrected phase shift. The TT and %BF were obtained per segment. In addition, the phase shift values per individual frequency bin (i.e., per 0.01 Hz frequency bin) were obtained. The latter was used to construct the TFA phase plot (0.01–0.5 Hz) and stored for later use (see Section 2.4.2).

Box 1 **NIRS-only methodology**

In the NIRS-only methodology, the transfer function analysis (TFA) phase shifts between oxyhemoglobin (oxyHb) and deoxyhemoglobin (deoxyHb) are corrected for phase shifts caused by two physiological factors not related to cerebral autoregulation (CA) effects. First, a constant microvascular transit time (TT blood flow, referred to as TT in the main text) effect, resulting in different phase shifts for different frequencies (‘group delay phenomenon’). Second, the ‘washout effect’, expressed as the ratio between slow changes in blood flow (BF) and blood volume (BV), results in phase shifts (BV expressed as the percentage of BF oscillations, %BF) [9].

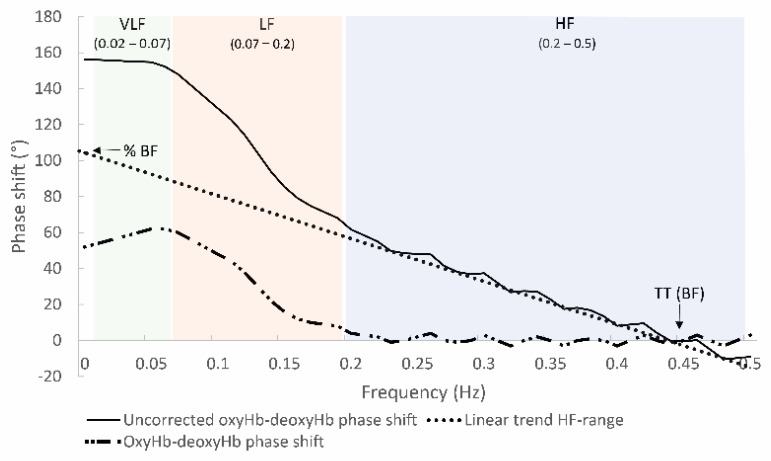

**Intact CA**
TFA-phase shift plot showing the high-pass filter principle (i.e., higher phase shift for lower frequencies). An example is shown here in the figure.
**Impaired CA**
TFA-phase shift plot showing low phase shifts (around zero) for all frequencies (i.e., ‘flat line’, not shown in this figure).
Schematic representation of the TFA phase shift plot. The different curves show (1) the uncorrected oxyHb—deoxyHb phase shift (solid line), (2) the best linear fit on the phase shifts in the high frequency (HF) range, called HF trend line (dotted straight line), and the corrected oxyHb—deoxyHb phase shift (dashdotted line). Phase shift correction is performed by subtracting the extrapolated linear HF trend line from the uncorrected phase shifts over the very low frequency (VLF) and low frequency (LF) ranges. %BF is determined by the y-axis intercept of the linear HF trend line.* The TT(BF) is determined by the x-axis intercept of the linear HF trend line. *
* calculations described in Elting et al. [9].

#### 2.4.2. Selection of Transfer Function Analysis (TFA) Segments

In addition to the CARNet TFA recommendations for data processing, we formulated criteria to select ten-minute TFA phase shift data segments (referred to as segments in the remaining text) based on the assumptions given in Table 1. Because segment selection might entail the risk of selection bias, we compared common physiological ICU parameters and the mean PSD of ABP and NIRS between the ‘in’- and ‘excluded’ segments. After segment selection, for the ‘included’ segments the model output variables, the mean ABP values and PSD values (ABP, oxyHb and deoxyHb) of the ‘included’ segments were averaged per patient and per hemisphere (with standard deviations (SD) as a measure for within-patient variability). In addition, other physiological data (like HR and EtCO_2_) were averaged per patient over the entire recording period (i.e., without data segment selection and artifact removal) (See Supplementary File S2).

### 2.5. Statistical Analysis

Data are presented as median and interquartile range (q1–q3) for continuous variables and frequencies (%) for categorical variables. First, we described the cohort including the number of ‘included’ and ‘excluded’ segments. Second, we described and compared clinical outcome groups regarding patient demographics, clinical variables, NIRS data length and data quality. Third, we compared ‘included’ and ‘excluded’ segments regarding signal characteristics (i.e., mean PSD results (oxyHb, deoxyHb and ABP) and physiological variables). Fourth, we compared clinical outcome groups regarding CA estimates and PSD. Fifth, we repeated the analysis for patients with a primary neurological admission diagnosis. Each patient was represented by the hemisphere with the worst CA—which was defined by the lowest LF-phase shift—in case bilateral NIRS measurements were available. 

The statistical relationship between dichotomized clinical outcome and the LF-phase shift was evaluated using the Mann–Whitney *U* test. To assess the influence of potential confounders, multivariable logistic regression was performed. Although no effect of age on CA phase shift has been found in the literature [15], age was included, being a strong predictor of outcome after ICU admission. Six-month clinical outcome was used as the dependent variable and the variables age, acute physiology and chronic health evaluation (APACHE) IV score and measurement time after ICU admission as independent variables. A *p*-value < 0.05 was considered as statistically significant. All statistical analyses were performed in R (version 4.0.3; R Core Team, Vienna, Austria) [16].

## 3. Results

**Figure 1:**
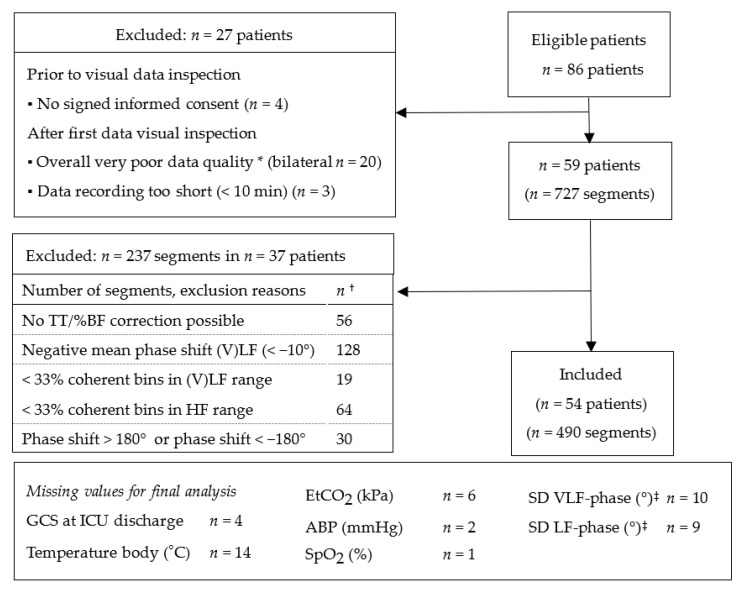
Study flow chart. Each box (right side) shows the remaining number of patients and remaining number of segments. For each patient a different number of segments is available. * Overall poor data quality e.g., crosstalk, no beat-to-beat pulsatility, multiple-artifacts. ^†^ The numbers do not count to the number of ‘excluded’ segments, because each segment can have more than one reason for exclusion. ^‡^ No SD could be calculated, as the patient is represented by only one segment. ABP = arterial blood pressure; BF = blood flow oscillations; EtCO_2_ = end tidal carbon dioxide; GCS = Glasgow coma scale; HF = high frequency range; (V) LF = (very) low frequency range; SD = standard deviation; SpO_2_ = peripheral oxygen saturation; TFA = transfer function analysis; TT = microvascular transit time.

We measured eighty-six critically ill sedated/comatose patients with a variety of admission diagnoses (Supplementary Table S3). The patient and segment selection flowchart is shown in Figure 1. For four patients no informed consent was given. We excluded the whole recordings of 23 patients during the first period of our study due to practical and technical issues (mainly crosstalk between the NIRS optodes, details in Figure 1). This led to a protocol change to avoid crosstalk. Fifty-nine patients with 727 segments were available for the TFA. Application of the (automated) selection of segments, resulted in 490 segments (67%) in fifty-four patients. On average four segments (q1–q3 2–7) were available per patient. The final study population consisted of predominantly middle-aged male patients of whom 63% (*n* = 34) had a primary neurological diagnosis (Table 2). The six-month mortality rate was 46% (*n* = 29 survivors versus *n* = 25 non-survivors). Six patients died between ICU discharge and planned follow-up. The survivors were on average younger compared to the non-survivors (49 (q1–q3 40–57) versus 71 (q1–q3 59–77) years old), had lower APACHE IV scores (65 (q1–q3 41–94) versus 102 (q1–q3 72–120) and were measured later (44 (q1–q3 20–84) versus 22 (q1–q3 13–45) hours) after ICU admission (Table 3). There were no clinically relevant physiological differences between the groups during the measurement period (Supplementary Table S4).

### 3.1. Selection of Transfer Function Analysis (TFA) Segments

Signal characteristics between ‘included’ segments (*n* = 490 in 54 patients) and ‘excluded’ segments (*n* = 237 segments in 37 patients) are compared in Supplementary Table S5. The percentage of raw oxyHb and deoxyHb signal artifact removal was similar between the groups (‘included’ 8.2% (q1–q3 1.4–17) versus ‘excluded’ 9% (q1–q3 2.3–30) segments). Both the mean PSD-oxyHb LF and PSD-ABP LF were higher for the ‘included’ segments (‘included’ PSD-oxyHb LF 0.03 (q1–q3 0.01–0.076) versus ‘excluded’ 0.012 (q1–q3 0.005–0.027) μM^2^/Hz; mean PSD-ABP LF (‘included’ 3.9 (q1–q3 1.0–14) versus ‘excluded’ 1.6 (q1–q3 0.4–3.6) mmHg^2^/Hz segments). There were no differences for the other physiological variables.

### 3.2. Cerebral Autoregulation (CA) Parameters

**Figure 2:**
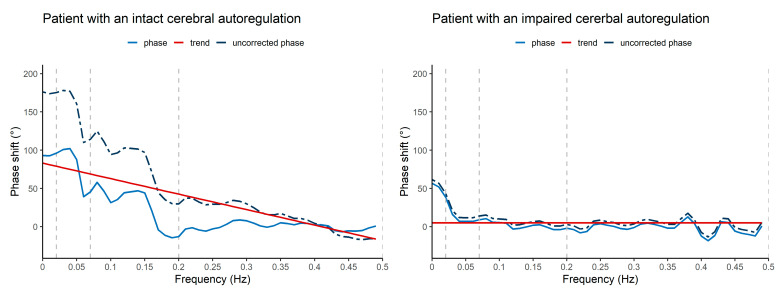
TFA-phase shift plot examples in two patients (ten-minute segment) of intact/impaired CA estimations. (**A**) shows a segment with a ‘high pass’ filter concept configuration and TFA VLF and LF numbers assuming working CA (mean LF-phase shift 19°). (**B**) shows a segment with a ‘flat line’ with minimal HF correction. No ‘high-pass’ filter principle configuration is seen and VLF and LF-phase shift numbers are close to zero (mean LF-phase shift 2°), assuming impaired CA. The dashed dark blue curve is the uncorrected phase shift computed for the frequency range 0.01–0.5 Hz. The lighter blue line is the phase shift computed after subtraction of the red linear HF trend line from the uncorrected phase shift. The dashed vertical grey lines delineate the frequency ranges for the VLF (0.02–0.07 Hz), LF (0.07–0.2 Hz) and HF (0.2–0.5 Hz) range. CA = cerebral autoregulation; deoxyHb = deoxyhemoglobin; HF = high frequency range; LF = low frequency range; oxyHb = oxyhemoglobin; TFA = transfer function analysis; VLF = very low frequency range.

**Figure 3:**
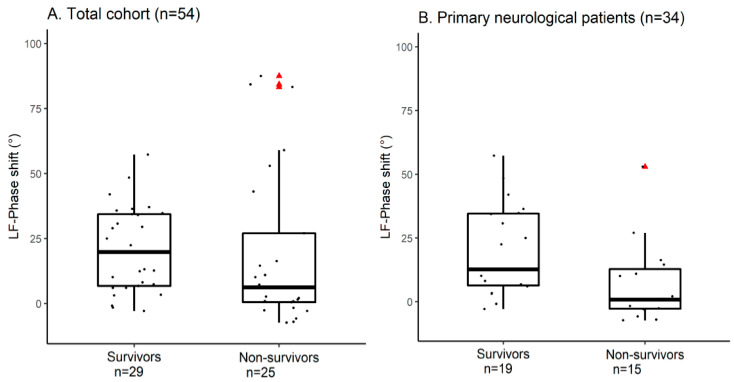
Low frequency phase shift dichotomized for six-month mortality. The boxplots in (**A**) show for the total cohort no significant difference between survivors and non-survivors for the LF-phase shift (survivors 20° (q1–q3 6.7–34) versus non-survivors 6.2° (q1–q3 0.51–27), *p* = 0.118, *n* = 54). Three outliers (∆ in the non-survivor group (LF-phase shift > 75°) were identified. These three patients had the admission diagnosis: respiratory insufficiency (*n* = 2) and hemorrhagic shock (*n* = 1). (**B**) shows the boxplots for patients with a primary neurological admission diagnosis. A significant difference between survivors and non-survivors for the LF-phase shift (survivors 13° (q1–q3 6.3–35) versus non-survivors 0.83° (−2.8–13), *p* = 0.0167, *n* = 34) is presented. The outlier (∆ in the non-survivor group (LF-phase shift = 52°) was a multi-trauma patient with only mild brain injury involvement who was intubated because of respiratory insufficiency. deoxyHb = deoxyhemoglobin; LF = low frequency range; oxyHb = oxyhemoglobin; q1–q3 = interquartile range; ∆ = data outlier.

Figure 2 shows two examples of the TFA phase shift plot of a segment: one segment with the interpretation of an intact CA (‘high-pass filter’ configuration and LF-phase shift of 19°, Figure 2A) and one segment with the interpretation of an impaired CA (almost ‘flat line’ configuration and LF-phase shift of 2°, Figure 2B). The median LF-phase shift of the study population was 12° (q1–q3 2.2–34). The coherence, remaining model output variables and the mean PSD results are summarized in Supplementary Tables S6 and S7. The TFA LF-phase shift values dichotomized for six-month mortality are given in Figure 3A. There was no significant difference between survivors and non-survivors (survivors 20° (q1–q3 6.7–34) versus non-survivors 6.2° (q1–q3 0.51–27), *p* = 0.118, *n* = 54). The within-patient LF-phase shift variability is shown in Supplementary Figure S4. Median LF-phase shift SD was 6.5° (q1–q3 3.5–14) with comparable results between the groups (survivors 8.3° (q1–q3 4–15) versus non-survivors 5.9° (q1–q3 2.9–9.3)).

### 3.3. Primary Neurological Admission Diagnosis

Thirty-four patients had a primary neurological admission diagnosis. The physiological and CA measures are described in Tables S8–S12. The LF-phase shift was significantly higher in survivors (survivors 13° (q1–q3 6.3–35) versus non-survivors 0.83° (q1–q3 2.8–13), *p* = 0.0167, Figure 3B), indicating a more preserved CA in survivors compared to non-survivors. This relationship remained significant after correction for age, APACHE IV score and measurement time after ICU admission in the multivariate model. Per 10° lower phase shift a significant increase in mortality was found (adjusted Odds Ratio (OR) of 0.27, 95%—confidence interval (95%-CI) 0.10–0.78, *p* = 0.015)). For the variable age (keeping the other predictors constant) a significant positive relationship with mortality was found (adjusted OR of 1.16, 95%-CI 1.01–1.33, *p* = 0.032). No significant independent relationship with mortality was found for APACHE IV score (*p* = 0.614) and measurement time after ICU admission (*p* = 0.218) (Supplementary Table S13).

## 4. Discussion

In this prospective single center observational study, we tested for the first time in critically ill and sedated/comatose patients our non-invasive methodology that uses high frequency cerebral oxyHb and deoxyHb NIRS signals to estimate CA. We upgraded the methodology by adding criteria to select segments for more robust and consistent CA estimations in individual patients. An estimation of CA could be provided in more than 90% of our patients (*n* = 54) with acceptable within-patient variability of the LF-phase shift, leaving around forty minutes of data available for the analysis per patient. Even after adoption of our bedside measurement protocol in 2019, 33% of the segments had to be excluded. In 34 patients with a primary neurological admission diagnosis, a significant and independent relationship with six-month mortality was found.

### 4.1. Data segment Selection

Although in this ‘NIRS-only’ methodology no continuous ABP recordings are required, sufficient hemodynamic oscillations are needed to challenge the cerebrovascular network. Although many maneuvers—like thigh cuffs or squad stands—have been tested successfully to induce ABP oscillations, they can be dangerous or impractical for critically ill patients [17]. Although CA-estimates that correlated with clinical outcome have been calculated in the time domain using spontaneous ABP oscillations, these measurements required long recordings and ABP-monitoring [18,19]. Data from multiple research sites suggested that when using TCD recordings of around five minutes, a mean PSD ABP LF of at least 10 mmHg^2^/Hz is required for reliable frequency domain CA estimations in healthy awake subjects [10]. Comparing the ‘in’- and ‘excluded’ segments in our study population supports that limited spontaneous ABP oscillations might explain periods with poor signal-to-noise ratio in our sedated patients and warrants the need for additional criteria for data selection. Being a ‘NIRS-only’ methodology, we formulated NIRS-based criteria making use of (1) the TFA-based ‘high-pass filter’ characteristic of dynamic CA and (2) the applied correction for serial time shifts between the oxyHb and deoxyHb signals through the capillaries (Box 1, Table 1). The CA concept can be described as a ‘high-pass’ filter with active altering of the lower frequency oscillations in the cerebral signals [8,20]. Most TFA CA papers report averaged values per frequency range. We decided to construct the TFA phase plot of all segments and reviewed from these plots the interpretability of the obtained phase shift values in the VLF, LF and HF range. We translated this into a set of criteria that can be applied in an automated way (Supplementary File S1).

### 4.2. Clinical Interpretation

The advantage of our ‘NIRS-only’ methodology is that it provides an easy way to measure CA that can be applied in different clinical situations (hospital wards, operating room but also outpatient clinic). In the current study, we evaluated the methodology in a critically ill sedated/comatose ICU population. Several studies showed that impaired CA in critically ill patients is related to poor clinical outcome [1,21]. We were able to replicate these findings for 34 patients with a primary neurological patient admission diagnosis. Interestingly, for the whole ICU study population, the LF-phase shift was low (12° (q1–q3 2.2–34)). So far, our ‘NIRS-only’ methodology has been applied in only two small studies [9,22]. In eleven healthy subjects, the baseline LF-phase shift was between 30° and 40° and decreased to values around 20° during hypercapnia. In a sepsis model in eleven healthy subjects, the baseline LF-phase shift values of 16.2° (q1–q3 3.0–52.6) decreased to 3.9° (q1–q3 2.0–8.8) after endotoxin infusion. Zhang et al. obtained the CA status in neurological ICU patients using ABP and TCD recordings and found an average LF-phase shift of about 30° in twenty patients [12]. This might suggest that in our critically ill and sedated/comatose population with high disease severity scores, the CA status was somehow impaired in general. Another factor that might explain the lower LF-phase shift values is, that the patients in our study were represented by the ‘worst’ CA hemisphere.

### 4.3. Limitations

Our study has several limitations that need to be addressed. Although the NIRS technique is easy to apply on the forehead, there are several issues that limits its application (even in sedated/comatose patients). Firstly, artifacts and crosstalk in particular (due to simultaneous, bilateral measurements). Unique for our NIRS device compared to other commercially available devices is that it collects high frequency raw data of 50 Hz. Noisy signals or artifacts are therefore clearly visible in the pulsating oxyHb and deoxyHb signals. For example, we noticed an artifact (of ~4.5 Hz) on top of the per heart-beat oscillations which was explained by crosstalk between the optodes. This artifact disturbed the TFA due to its high amplitude, and broad and varying frequency content. For this reason, we had to exclude whole patient recordings in a large number of patients and had to change our measurement protocol from bilateral, simultaneous to unilateral, consecutive measurements. The latter limits the comparison of both hemispheres over time. Regarding the crosstalk, incorporating asynchronous data sampling can solve the crosstalk problem. The influence of the pre-processing (artifact removal) was not evaluated formally, but it is recommended to remove artifacts (preferably in an automated way), as this results in incorrect high coherence values between both NIRS signals [8].

Secondly, there is controversy about the exact measurement depth of the NIRS technology and the degree to which the signals are influenced by skin or bone tissue [23]. We used the deepest recording loop of our device, but without the option to correct for more superficial light absorption.

Thirdly, we did not use another (non-invasive) brain monitoring technique to compare our results with. TCD is often used as a reliable technique to estimate CA in the frequency domain, but usually allows only short recordings. Our ‘NIRS-only’ methodology was previously compared with TCD results in healthy subjects which is reassuring [9], but the recent modifications (Supplementary Table S2) and data segment selection option justify a new comparison. In addition, we did not compare our results with a model including ABP signals. A NIRS model using a slightly different HF-range correction for serial time trends was previously studied in awake patients with (mild) cognitive and in healthy subjects. They studied the relationship between ABP and oxyHb [24]. It might be interesting to compare our results with those retrieved from ABP—oxyHb calculations.

Fourthly, our outcome analysis and results are hampered by the short recordings (forty minutes on average), the high mortality rates and the majority of patients having a primary neurological admission diagnosis. This limits the generalizability of our findings. In addition, the outcome analysis is likely affected by other pathophysiological processes during the ICU stay. However, the relationship of LF-phase shift with outcome in primary neurological patients was independent from disease severity, age and measurement time.

Fifthly, we experienced difficulties with some digital signal processing steps. As an example, two of the data selection criteria are directly related to the phase wrap around phenomenon. Phase wrap around is a complex data processing problem in TFA phase shift calculations, as for phase calculations the inverse tangent is computed and the inverse tangent cannot differentiate between π (180°) and 2π (360°) radians. Therefore, CARNet recommends removing phase shift values showing wrapped phases [8]. In our software we applied methods to adjust automatically for phase wrap around (Supplementary Table S2), but phase wrap was not always recognized. We therefore restricted the phase shift values to the range [−180°–180°] and removed mean negative (V)LF phase shift values (<−10°) computed per segment [8]. The arbitrary −10° threshold was chosen to accept small calculation errors for patients with total CA impairment. Another example that likely has affected our data is the effect of mechanical ventilation on our applied BF/BV and TT correction, as the ventilation rate is within the HF range. The positive pressure ventilation influenced the HF trend line estimation in some segments quite dramatically. Future studies should investigate more in-depth into the effect of mechanical ventilation on phase shifts between oxyHb and deoxyHb and on our ‘NIRS-only’ CA methodology.

Sixthly, the unexpected difference between the groups at the start of the measurement might have resulted from a recruitment selection bias. However, in our multivariate outcome model we corrected for this potential confounder (Supplementary Table S13).

Lastly, the measurement timing for nineteen patients was outside our intended 48 h window (35%) after ICU admission. Although this might have affected our results, for a feasibility study this protocol violation might be of less importance.

### 4.4. Future Perspectives

Our results show that the ‘NIRS-only’ methodology seems applicable on the ICU when taking data processing, data selection criteria and limitations of the NIRS technology into account. Future studies are required for the validation of our modifications and selection criteria in larger study populations and should investigate more in-depth the effect of certain interventions like mechanical ventilation on the CA estimation. The methodology should also be tested with signals from other available NIRS devices. Furthermore, the performance of our methodology should be investigated in longer recordings to monitor the CA status over time.

## 5. Conclusions

CA estimation using the ‘NIRS-only’ methodology seems feasible in critically ill sedated/comatose patients after incorporation of methodological improvements and automated data segment selections for more robust and consistent CA estimations. We found an independent and significant correlation between LF-phase shift and six-month mortality in patients with a primary neurological admission diagnosis. CA estimation without the need for continuous ABP measurement (‘NIRS-only’ methodology) is an attractive option to be (continuously) informed about the individual CA status.

## Figures and Tables

**Table 1:** Transfer function analysis phase plot criteria for segment selection. A detailed rationale for the automated ten-minute TFA phase plot data segment selection (segment) is provided in Supplementary File S2.

Assumptions	Criteria to Exclude a Segment	Reference
(I) The physiological high-pass filter characteristics of CA are observed in the VLF and LF range; (II) Reliable correction for serial time effects using the HF data (correction for TT and %BF) is performed [9].	Correction for TT and %BF was not possible, i.e., no HF trend line was available.	[9], Box 1, Supplementary Figure S2, Supplementary Table S2
	A negative mean VLF and/or LF-phase shift of <−10° was present.	[8]
	Mean VLF and/or LF-phase shift values of >180° or <−180° (likely caused by persistence of ‘phase wrap around’) were present.	[8], Supplementary Table S2
	<33% of the frequency bins in the VLF + LF or the HF-range had a coherence value above the significance threshold (meaning <6 bins available for the VLF + LF range and <10 bins for the HF range).	Figure S3

%BF = percentage blood flow oscillations; CA = cerebral autoregulation; HF = high frequency; LF = low frequency; TT = microvascular transit time; TFA = transfer function analysis; VLF = very low frequency.

**Table 2:** Patient characteristics dichotomized for six-month mortality.

Median (q1–q3)	Total (*n* = 54)	Survivors (*n* = 29)	Non-Survivors (*n* = 25)
Age (years)	58 (43–72)	49 (40–57)	71 (59–77)
Sex, male, *n* (%)	46 (85)	22 (76)	24 (96)
Admission diagnosis, primary neurological *, *n* (%)	34 (63)	15 (52)	19 (76)
Admission APACHE IV score	84 (51–111)	65 (41–94)	102 (72–120)
SOFA score (on day of measurement)	9 (6–10)	8 (6 -10)	10 (7–11)
Length of ICU stay (days)	12 (6–17)	15 (6.2–20)	8.4 (5.9–15)
Days on mechanical ventilation	7.2 (2.9–12)	8.5 (2.9–14)	7.2 (3.6–11)
Mortality at ICU discharge, *n* (%)	19 (34)	0	19 (76) ^‡^
GCS at ICU discharge ^†^, *n (%)*			
GCS score 3–5	1 (1.9)	0	1 (4)
GCS score 6–8	1 (1.9)	0	1
GCS score 9–12	8 (15)	7 (25)	1
GCS score 13–15	21 (39)	19 (72)	2
GOSE at 6 months, *n* (*%*)			
Favorable outcome, GOSE 5–8	25 (46)	25 (86)	0
Unfavorable outcome,	4 (7.4)	4 (14)	0
GOSE 2–4			
Mortality GOSE 1	25 (46)	0	25 (100)

* Primary neurological diagnoses include: traumatic brain injury, cardiac arrest, acute stroke, status epilepticus and meningitis (see Supplementary Tables S4–S8). ^†^ The number of missing GCS at ICU discharge values are for survivors *n* = 3 and non-survivors *n* = 1. ^‡^ Six patients died between ICU discharge and six months follow-up. Admission diagnoses of these patients were: acute stroke (*n* = 2), respiratory insufficiency (*n* = 3) and hemorrhagic shock (*n* = 1). APACHE IV = acute physiology and chronic health evaluation IV; GCS = Glasgow coma scale; GOSE = Glasgow outcome scale extended; ICU = intensive care unit; SOFA= sequential organ failure assessment; TBI= traumatic brain injury.

**Table 3:** Near infrared spectroscopy data length and quality dichotomized for six-month mortality. The results of the unilateral hemispheric measurement are reported, i.e., the hemisphere with the worst cerebral autoregulation estimate (lowest LF-phase shift for an individual).

Median (q1–q3)	Total (*n* = 54)	Survivors (*n* = 29)	Non-Survivors (*n* = 25)
Bilateral measurements, *n* (*%*)	40 (74)	19 (54)	21 (84)
Start measurement after ICU admission (h)	29 (16–77)	44 (20–84)	22 (13–45)
Duration bedside recording (min)	77 (59–130)	71 (68–59)	79 (63–125)
Artifact free NIRS recording (min) *	61 (47–121)	54 (53–46)	67 (48–122)
NIRS data removed ^†^ (%)	9.5 (2–26)	11 (2.9–27)	8.8 (1.6–17)
Number of segments per patient *	4 (2–7)	4 (2–6)	5 (2–7)

* Discrepancy between artifact free NIRS recordings and number of ten-minute TFA phase plot segments is due to the requirement of ten contiguous minutes to be selected as a data segment. ^†^ The removed NIRS data (before data processing) as a percentage of the recorded data. LF = low frequency; ICU = intensive care unit; NIRS = near infrared spectroscopy; q1–q3 = interquartile range; TFA = transfer function analysis.

## Data Availability

Data is contained within the article or supplementary material.

## Supplementary Materials

The following supporting information can be downloaded at: https://www.mdpi.com/article/10.3390/cells11142254/s1. Table S1: STROBE Statement—checklist of items that should be included in reports of observational studies; File S1: Extra information about the applied Methodology; Figure S1: Artefact removal algorithm; Table S2: ‘NIRS-only’ methodology modifications with respect to Elting et al. [1]; Figure S2: Examples of linear polynomial high frequency range fit; File S2: Transfer function analysis phase shift plot segment selection; Figure S3: Threshold for the number of coherent bins; Table S3: Patient clinical admission diagnosis dichotomized for six-month mortality; Table S4: Physiological variables during the bedside measurement dichotomized for six-month mortality; Table S5: Characteristics of ‘included’ versus ‘excluded’ segments; Table S6: Power spectral density results dichotomized for six months mortality; Table S7: Cerebral autoregulation parameters (TFA estimates) results dichotomized for six-month mortality; Table S8: Patient characteristics dichotomized for six-month mortality for primary neurological diagnosis; Table S9: NIRS data length and quality dichotomized for six-month mortality for primary neurological diagnosis; Table S10: Frequency analysis dichotomized for six-month mortality for primary neurological diagnosis; Table S11: Cerebral autoregulation parameters dichotomized for six-month mortality for primary neurological diagnosis; Table S12: Physiological variables during the bedside measurement dichotomized for six-month mortality for primary neurological diagnosis; Figure S4: Within patient variability for the low frequency phase shift (*n* = 54); Table S13: Multivariate logistic regression model for primary neurological diagnosis (*n* = 34). References [25,26] are cited in the supplementary materials.
