# Supplementary material for: Cerebral Autoregulation Assessment Using the Near Infrared Spectroscopy ‘NIRS-Only’ High Frequency Methodology in Critically Ill Patients: A Prospective Cross-Sectional Study
Source: Cells. 2022 Jul 21;11(14):2254. doi: 10.3390/cells11142254 (PMC9317651; doi:10.3390/cells11142254)
Supplement: Supplementary file 1 [file cells-11-02254-s001.zip › cells-1786525-supplementary.pdf]

Cerebral autoregulation assessment using the near infrared spectroscopy 'NIRS-only' high frequency methodology in critically ill patients: A prospective cross-sectional study

Jeanette Tas,[1,2*,†] Nick Eleveld,[3,†] Melisa Borg,[1] Kirsten D.J. Bos,[1] Anne P. Langermans,[1] Sander M.J. van Kuijk,[4] Iwan C.C. van der Horst,[1,5] Jan Willem J. Elting,[3,‡] Marcel J.H. Aries[1,2,‡]

[†] These authors contributed equally to this work.

[‡] These authors contributed equally to this work.

* Correspondence: tasjeanette@gmail.com.

[1] Department of Intensive Care Medicine, University Maastricht, Maastricht University Medical Center+, Maastricht, The Netherlands

[2] School for Mental Health and Neuroscience (MHeNS), University Maastricht, Maastricht, The Netherlands

[3] Department of Neurology and Clinical Neurophysiology, University Medical Center Groningen, Groningen, The Netherlands

[4] Department of Clinical Epidemiology and Medical Technology Assessment, (KEMTA), Maastricht University Medical Center+, Maastricht, The Netherlands

[5] Cardiovascular Research Institute Maastricht (CARIM), Maastricht, The Netherlands

**Table S1**	STROBE Statement - checklist of items that should be included in reports of observational studies
**Supplementary File S1**	Extra information about the applied Methodology
**Figure S1**	Artefact removal algorithm
**Table S2**	‘NIRS-only’ methodology modifications with respect to Elting et al. (2018)
**Figure S2**	Examples linear polynomial high frequency range fit
**Supplementary File S2**	Transfer function analysis phase shift plot segment selection
**Figure S3**	Threshold for the number of coherent bins
**Table S3**	Patient clinical admission diagnosis dichotomized for six-month mortality
**Table S4**	Physiological variables during the bedside measurement dichotomized for six-month mortality
**Table S5**	Characteristics of ‘included’ versus ‘excluded’ segments
**Table S6**	Power spectral density results dichotomized for six months mortality
**Table S7**	Cerebral autoregulation parameters (TFA estimates) results dichotomized for six-month mortality.
**Table S8**	Patient characteristics dichotomized for six-month mortality for primary neurological diagnosis
**Table S9**	NIRS data length and quality dichotomized for six-month mortality for primary neurological diagnosis
**Table S10**	Frequency analysis dichotomized for six-month mortality for primary neurological diagnosis
**Table S11**	Cerebral autoregulation parameters dichotomized for six-month mortality for primary neurological diagnosis
**Table S12**	Physiological variables during the bedside measurement dichotomized for six-month mortality for primary neurological diagnosis
**Figure S4**	Within patient variability for the low frequency phase shift (*n* = 54)
**Table S13**	Multivariate logistic regression model for primary neurological diagnosis (*n* = 34)

Supplementary File S1. Extra information about the applied Methodology

1 Data collection

For each patient, we collected the following patient and admission characteristics: age, sex, admission diagnosis, acute physiology and chronic health evaluation IV (APACHE IV) score at admission, sequential organ failure assessment (SOFA) score on the measurement day, ICU length of stay and the number of days on mechanical ventilation. Close to the measurement, the following variables were retrieved from laboratory or arterial blood gas analysis results: partial oxygen tension (PaO_2, kPa), partial carbon dioxide tension ($PaCO_2$, kPa), the PaO_2 / FiO_2 ratio (mmHg/%), and hemoglobin concentrations (mM). In addition, the Glasgow coma scale (GCS) at ICU discharge (from discharge letter or nursing chart), the ICU mortality (from discharge letter), and the six-month Glasgow outcome scale extended (GOSE) by telephone interview were collected as clinical outcome parameters.

2 Arterial blood pressure monitoring

The arterial blood pressure (ABP) was monitored invasively in the arteria radialis or femoralis and in most patients zeroed at (right) heart level. In traumatic brain injury (TBI) patients with intracranial pressure monitoring, the ABP was zeroed at the foramen of Monroe level to be able to calculate the cerebral perfusion pressure

3 High frequency NIRS monitoring

The Portalite NIRS has an emitter of infrared (IR) light and three receivers for the IR light. The distance between the emitter and the receivers determines the depth that the light transfers through the (brain)tissue. The Portalite measures at three distances (superficial, intermediate, and deepest loop). We used the deepest loop (largest inter-optode distance of 40 mm) for our analyses. The largest inter-optode distance increased the likelihood of measuring brain tissue. Age-related pathlength scattering correction was applied for the age range 17-50 years [1]. In contrast to other commercial NIRS devices, the Portalite collects high-frequency (50Hz) data. For the 'NIRS-only' methodology, a NIRS device with oxyhemoglobin (oxyHb) and deoxyhemoglobin (deoxyHb) signals with at least a sampling frequency of 1 Hz is needed so frequencies up to 0.5 Hz can be reliably measured.

Via a custom-made ICM+ Portalite module, these high frequency NIRS data were transferred in real-time from the NIRS recording Oxysoft software (version: 3.0.103.3; Artinis Medical Systems, Elst, The Netherlands) to the ICM+ software.

4 Power spectral density analysis

Sufficient slow ABP fluctuations are required for a reliable CA assessment [2]. The amount of slow ABP, oxyHb and deoxyHb fluctuations was quantified by the Power Spectral Density (PSD) over detrended ABP, oxyHb, deoxyHb-traces using Welch's method: 100 second windows, 50% window overlap, and a Hanning window. The ABP signals were pre-processed automatically with the removal

of segments with ABP values < 0 mmHg, systolic ABP values > 200 mmHg, or data gaps > 0.2 sec (mainly caused by zeroing, flushing, or manipulation of the arterial line). After that, the mean PSD for the very low frequency (VLF) and low frequency (LF) range for ABP, oxyHb, and deoxyHb was determined per patient.

5 Transfer function analysis

The relationship between oxyHb (input) and deoxyHb (output) in the different frequency ranges was studied by TFA [3]. The frequency ranges of interest are the very low frequency (VLF 0.02 - 0.07 Hz), the low frequency (LF 0.07 - 0.2 Hz), and the high frequency range (HF 0.2 - 0.5 Hz). From this analysis, the parameters coherence, gain, and phase shift are retrieved to represent different aspects of CA. In short, in the frequency domain, coherence is a measure of the linear relation between the signals (unitless), the gain is the amplification factor between the in-and output signals (unitless), and the phase shift is the time shift between the signals with the same frequency (in degrees (◦)) [4].

Table S1. Strengthening the Reporting of Observational studies in Epidemiology (STROBE) - checklist of items that should be included in reports of observational studies.

	Item No	Recommendation	Page No
**Title and abstract**	1	(*a*) Indicate the study's design with a commonly used term in the title or the abstract.	1
		(*b*) Provide in the abstract an informative and balanced summary of what was done and what was found.	1
**Introduction**			
Background/ rationale	2	Explain the scientific background and rationale for the investigation being reported.	1-2
Objectives	3	State specific objectives, including any prespecified hypotheses.	2
**Methods**			
Study design	4	Present key elements of study design early in the paper.	2
Setting	5	Describe the setting, locations, and relevant dates, including periods of recruitment, exposure, follow-up, and data collection.	2-3
Participants	6	(*a*) *Cohort study*—Give the eligibility criteria, and the sources and methods of selection of participants. Describe methods of follow-up<br>*Case-control study*—Give the eligibility criteria, and the sources and methods of case ascertainment and control selection. Give the rationale for the choice of cases and controls.<br>*Cross-sectional study*—Give the eligibility criteria, and the sources and methods of selection of participants.	2
		(*b*) *Cohort study*—For matched studies, give matching criteria and number of exposed and unexposed.<br>*Case-control study*—For matched studies, give matching criteria and the number of controls per case.	
Variables	7	Clearly define all outcomes, exposures, predictors, potential confounders, and effect modifiers. Give diagnostic criteria, if applicable.	3,4,5
Data sources/ measurement	8*	For each variable of interest, give sources of data and details of methods of assessment (measurement). Describe comparability of assessment methods if there is more than one group.	3 and 5
Bias	9	Describe any efforts to address potential sources of bias.	4
Study size	10	Explain how the study size was arrived at.	2
Quantitative variables	11	Explain how quantitative variables were handled in the analyses. If applicable, describe which groupings were chosen and why.	4 and 5
Statistical methods	12	(*a*) Describe all statistical methods, including those used to control for confounding.	5
		(*b*) Describe any methods used to examine subgroups and interactions.	5
		(*c*) Explain how missing data were addressed.	AP. A4
		Cross-sectional study—If applicable, describe analytical methods taking account of sampling strategy.	NA
		(*e*) Describe any sensitivity analyses.	NA

Continued on next page

**Results**			
Participants	13*	(a) Report numbers of individuals at each stage of study—eg numbers potentially eligible, examined for eligibility, confirmed eligible, included in the study, completing follow-up, and analysed	5-6
		(b) Give reasons for non-participation at each stage	5-6
		(c) Consider use of a flow diagram	6
Descriptive data	14*	(a) Give characteristics of study participants (eg demographic, clinical, social) and information on exposures and potential confounders	6-7
		(b) Indicate number of participants with missing data for each variable of interest	6
		(c) *Cohort study*—Summarise follow-up time (eg, average and total amount)	
Outcome data	15*	*Cohort study*—Report numbers of outcome events or summary measures over time	
		Case-control study—Report numbers in each exposure category, or summary measures of exposure	
		Cross-sectional study—Report numbers of outcome events or summary measures	*7*
Main results	16	(*a*) Give unadjusted estimates and, if applicable, confounder-adjusted estimates and their precision (eg, 95% confidence interval). Make clear which confounders were adjusted for and why they were included	8
		(*b*) Report category boundaries when continuous variables were categorized	7 and 8
		(*c*) If relevant, consider translating estimates of relative risk into absolute risk for a meaningful time period	NA
Other analyses	17	Report other analyses done - e.g. analyses of subgroups and interactions, and sensitivity analyses	8
**Discussion**			
Key results	18	Summarise key results with reference to study objectives	11
Limitations	19	Discuss limitations of the study, taking into account sources of potential bias or imprecision. Discuss both direction and magnitude of any potential bias	10-11
Interpretation	20	Give a cautious overall interpretation of results considering objectives, limitations, multiplicity of analyses, results from similar studies, and other relevant evidence	10
Generalisability	21	Discuss the generalisability (external validity) of the study results	10
**Other information**			
Funding	22	Give the source of funding and the role of the funders for the present study and, if applicable, for the original study on which the present article is based	12

*Give information separately for cases and controls in case-control studies and, if applicable, for exposed and unexposed groups in cohort and cross-sectional studies [5].

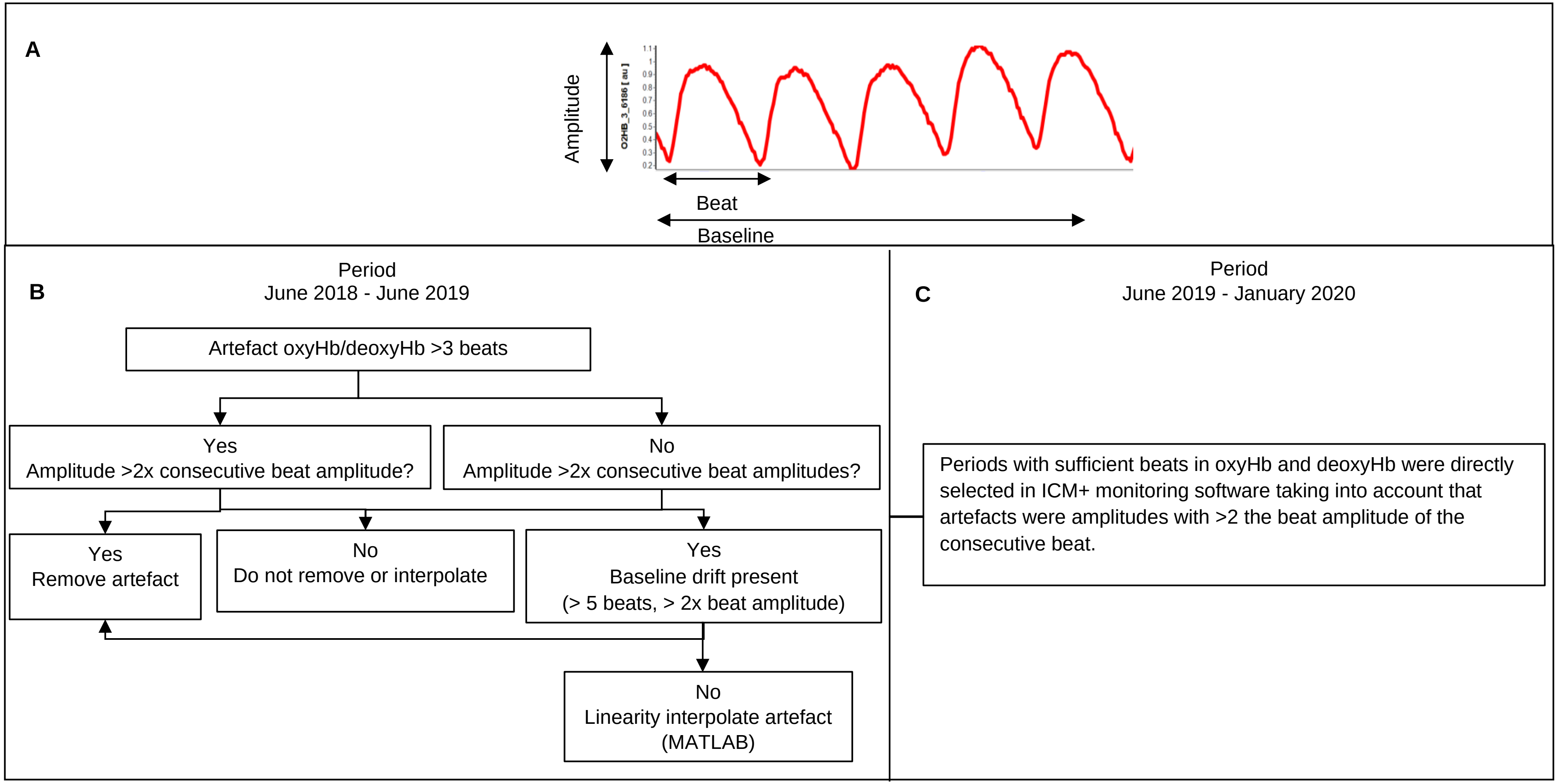

Figure S1. Artefact removal algorithm. (A) A period of five beats of the 50 Hz oxyHb signal. The beats are reflections of heart beats. Artefact removal was performed differently in the two measurement periods. **(B)** For the first period, sample numbers of artefacts in the oxyHb and deoxyHb signal were notated in a Microsoft Excel workbook by inspecting the imported data in LABVIEW. Subsequently, artefacts were removed or interpolated using the Excel workbook information in combination with a MATLAB algorithm. **(C)** In the second period (after the protocol change), periods with sufficient beats in the oxyHb and deoxyHb signals were selected directly in the monitoring software ICM+ taking into account the artefact criteria from period one. Artefacts in period 2 were the result of frequent ectopic heart beats in critically ill patients or manipulation of the optodes). deoxyHb = deoxyhemoglobin; oxyHb = oxyhemoglobin.

Table S2. 'NIRS-only' methodology modifications with respect to Elting et al. (2018)

Modification	Description
Automated Phase wrapping correction	In contrary to the 'manual' unwrapping correction in the previous model [6], the unwrapping was in the current study 'automated' with the inbuilt 'unwrap phase' function in LABVIEW. This function compares the phase shifts of two consecutive frequency bins. If the difference in phase shift is larger than 180°, 360° was added to the phase shift of the latter bin:<br>If $\lvert\phi_{n} - \phi_{n+1}\rvert > 180°$: $\phi_{n+1} \leftarrow \phi_{n+1} + 360°$ with ϕ_n = phase shift difference at position n and ϕ_{n+1} = phase shift difference at position n + 1 frequency bin.<br><br>However, if the first frequency bin was phase wrapped (i.e. ϕ is close to -180°), the phase shift in all the following frequency bins were incorrectly changed into values around -180°. To correct for this, 180°was added to the phase shifts in all frequency bins when then mean of the phase shifts was below a threshold of -90°.
Y intercept wrap limit	A setting to additionally correct for wrapped phases. Default setting:, -180°**.** This setting was functional in case the 'phase wrap limit mean' was not sufficient to correct for phase wrap. By using this setting, the calculations for TT(BF), %BF and y-intercept corrects also for the phase wrap
Slope min, Slope max, Intercept min, Intercept max,	Settings to add restrictions to the HF-trendline estimation. These settings can be set e.g. to avoid a HF-trendline with an unphysiological positive slope. Default settings: slope min: infinite, maximum: 0°, intercept minimum: 0°, intercept maximum: 180°).<br><br>For the current analysis, we additionally evaluated the effect of small positive slopes of the HF-trendline (to avoid too strict application of our segment selection). In case of small positive slopes, no phase shift correction was done and the uncorrected phase shift was provided. Small slopes were defined as a threshold < 0.3º /bin. This corresponds to a maximal change in phase shift of 9º. In case the HF-trendline slope of was larger than this threshold, no phase shift values were provided. Two examples of this applied criterion are given in Supplementary Figure S2.<br><br>When no HF-trendline correction was applied, the model assumes that BV oscillations dominates BF oscillations. Therefore, the TT(BF) cannot be defined, but assumed to be very close to zero (equation 4 in ref [6]).
BV = blood volume; BF = blood flow; HF = high frequency; TT (BF) = microvascular transit time (resulting from blood flow oscillations only).	

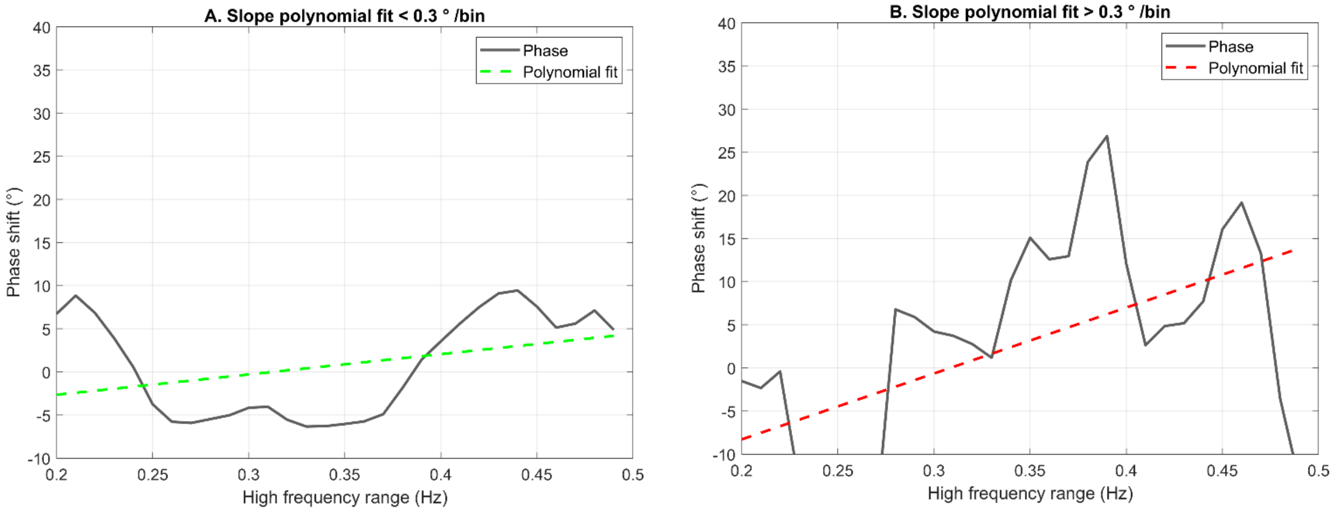

Figure S2. Examples of the linear polynomial high frequency range fit. The high frequency (0.2 - 0.5 Hz) range (x-axis) and the phase shift between oxyHb and deoxyHb concentration differences (y-axis). **(A)** This example shows the slope of the fitted linear polynomial less than 0.3^{o} /bin (uncorrected phase shift values for the (very) low frequency ranges were used) and **(B)** shows an example for which the slope of the linear polynomial increases with more than 0.3^{o} /bin (no phase shift values were provided. Segment was not used for outcome analysis). See further explanation in Supplementary Table S2.

Supplementary File S2. Transfer function analysis phase shift plot segment selection

The transfer function analysis (TFA) phase shift plots were computed over ten minute data segments (segments). The four selection criteria for physiologically interpretable TFA phase shift plots are described below. .

Criterion I. For the serial transit time - blood flow/blood volume (TT-BF/BV) correction, a linear fit over the phase shift values in the high frequency (HF)-range is used (HF-trendline). Segments with a significant positive slope for the linear HF-trendline were excluded (Supplementary Figure S2). The motivation is that a clear positive slope indicates a negative transit time between oxyhemoglobin (oxyHb) and deoxyhemoglobin (deoxyHb), which entails that oscillations in deoxyHb (mainly venous microvasculature) occur ahead of oscillations in oxyHb (mainly arterial microvasculature). We regard this as very unlikely. It is most probable that the significant positive slope is the result of motion artefacts and device noise, that affect the HF-trendline and no reliable correction in the methodology can be applied. Smaller positive HF-trendlines were treated differently (see Supplementary Table S2 and Supplementary Figure S2).

Criterion II. For the very low frequency (VLF)- and low frequency (LF)-range segments with negative mean phase shift results (< -10º) were excluded , This was applied to all VLF- and LF-range frequencies (< 0.2 Hz) for consistency, whereas removal of all negative phase shift values <0.1 Hz is recommended by The Cerebrovascular Research Network (CARNet) [3]. The occurrence of small negative phase shift values ($0 > \phi > -10^{\circ}$) was interpretated as impaired cerebral autoregulation.

Criterion III. Phase shift values above >180º or < -180º were excluded to account for wrap around that was not detected by our extensive automated algorithm. The removal of phases wrap around is in accordance with the CARNet recommendations [3]. (Supplementary Table S2 explains details about the automated phase wrap correction).

Criterion IV. A minimal number of bins with a coherence above the (calculated) significance threshold was determined. The smaller the number of allowed missing bins per frequency range (Supplementary Figure S3) , the larger the change in number of excluded segments. However, a more stringent threshold on the minimal number of allowed missing bins per frequency range naturally leads to the exclusion of more segments. Supplementary Figure S3 displayed the trade-off between minimal number of bins and number of excluded segments . A threshold of 66% was determined as this resulted in low exclusion of additional segments (5.8%, 30/520 segments), and yielded at least six bins for the VLF- plus LF-range and ten bins for the HF-range.

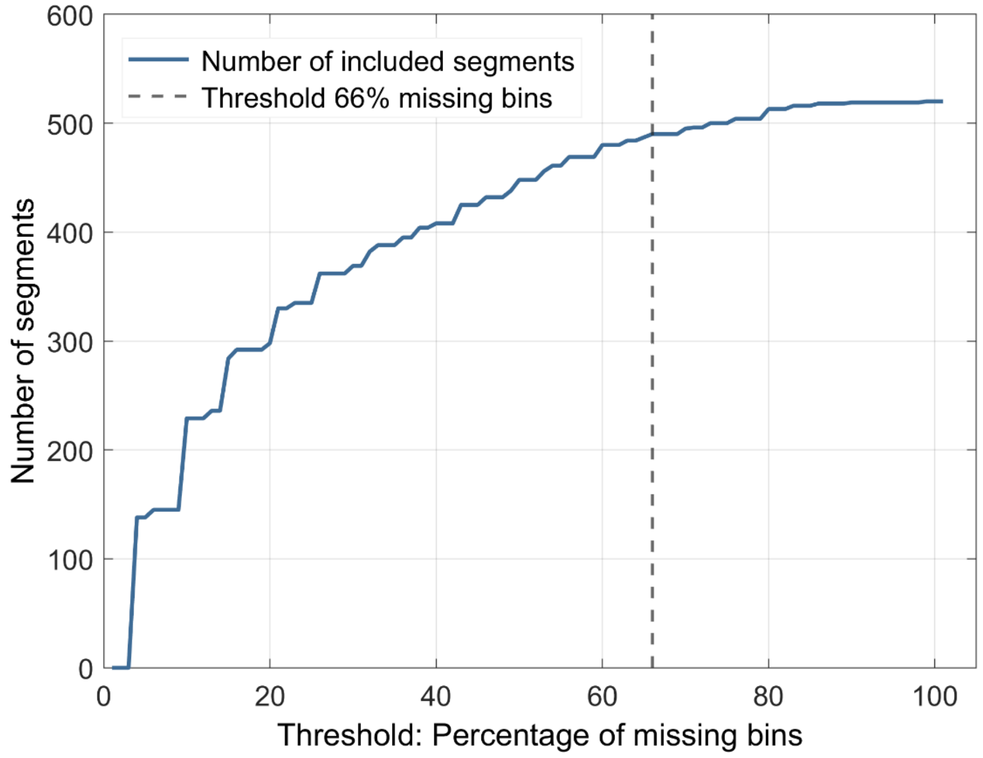

Figure S3. Threshold for the number of coherent bins. The trade-off between number of missing bins (%) (x-axis) and the number of included segments (y-axis). The graph shows the number of excluded segments after applying criteria I, II, III (Supplementary File S2). A higher threshold (x-axis) result in more included than excluded segments. The number of excluded segments for a threshold of 0% allowed no segments. A threshold of 66% (dotted line) is used, resulting in the inclusion of 490 of 520 segments.

Table S3. Patient clinical admission diagnosis dichotomized for six-month mortality

**Median (q1-q3)**	**Total (*n* = 54)**	**Survivors (*n* = 29)**	**Non-survivors (*n* = 25)**
Admission clinical diagnosis, n (%)			
Traumatic brain injury	13 (24)	10 (34)	3 (12)
Cardiac shock	1 (1.9)	1 (3.4)	0
Meningitis	1 (1.9)	1 (3.4)	0
Status epilepticus	1 (1.9)	0	1 (4)
Respiratory insufficiency	7 (13)	1 (3.4)	6 (24)
Acute Stroke	9 (17)	2 (6.9)	7 (28)
Post-surgical observation	2 (3.7)	1 (3.4)	1 (4)
Septic shock	2 (3.7)	2 (6.9)	0
Cardiac arrest	10 (19)	6 (21)	4 (16)
General (non-TBI) trauma	3 (5.6)	3 (10)	0
Hemorrhagic shock	4 (7.4)	1 (3.4)	3 (12)
Upper airway obstruction due to lymphoma	1 (1.9)	1 (3.4)	0
TBI = traumatic brain injury; q1-q3 = interquartile range			

Table S4. Physiological variables during the bedside measurement dichotomized for six-month mortality. The results of the unilateral hemispheric measurement period are reported, i.e. of the hemisphere with the worst cerebral autoregulation estimate (lowest LF-phase shift for an individual).

**Median (q1 - q3)**	**Total (*n* = 54)**	**Survivors (*n* = 29)**	**Non-survivors (*n* = 25)**
MAP (mmHg) – segments*	76 (71 - 85)	77 (71 - 84)	78 (70 - 88)
Heart rate (min^{-1})	79 (65 - 88)	82 (72 - 96)	77 (62 -85)
$EtCO_2$ (kPa) ‡	4.1 (3.7 - 4.7)	4.4 (4.0 - 4.8)	4.0 (3.5 - 4.3)
SpO_2 (%)§	97 (95 - 98)	97 (96 - 98)	96 (95 - 98)
Body temperature (°C) ǁ	36.8 (36.1 - 37.2)	37 (36.4 - 37.5)	36.7 (35.8 - 37.1)
FiO_2 (%)	35 (26 - 40)	30 (25 - 40)	40 (30 – 50)
During measurement			
PaO_2 (kPa)	11.6 (9.7 - 13.4)	11.5 (9.9 - 13.4)	11.6 (9.5 -13.2)
$PaCO_2$ (kPa)	4.9 (4.4 - 5.6)	5.1 (4.7 - 5.4)	4.9 (4.3 - 5.7)
PaO_2 / FiO_2 ratio (mmHg/%)	240 (187 - 328)	262 (225 - 337)	202 (142 - 270)
Hemoglobin (mM)	6.6 (5.7 - 8.2)	6.9 (5.9 - 8.1)	6.5 (5.7 - 8.2)

* The number of missing MAP values for non-survivors *n* = 2.
‡ The number of missing $EtCO_2$ values for survivors *n* = 2 and for non-survivors *n* = 4.
§ The number of missing SpO_2 values for survivors *n* = 1.
ǁ The number of missing body temperature values for survivors is *n* = 9 and for non-survivors *n* = 5.
$EtCO_2$ = end tidal carbon dioxide; FiO_2 = oxygen fraction; MAP = mean arterial blood pressure; $PaCO_2$= partial carbon dioxide pressure; PaO_2 = partial oxygen pressure; SpO_2 = peripheral oxygen; q1 - q3 = interquartile range.

Table S5. Characteristics of 'included' and 'excluded' segments

**Median (q1 - q3)**	**Total segments (n = 727)**	**'Included' segments* (n = 490)**	**'Excluded' segments* (n = 237)**
Physiological values			
MAP (mmHg) – segments†	78 (71 - 87)	77 (69 - 85)	78 (71- 87)
Heart rate (min^{-1})	80 (65 - 92)	80 (65 - 92)	79 (66 - 95)
$EtCO_2$ (kPa) §	4.1 (3.7 - 4.6)	4.2 (4.0 - 4.8)	4.0 (3.5 - 4.5)
During measurement			
PaO_2 (kPa)	11.9 (10 - 13.3)	11.9 (9.8 - 13.9)	11.6 (10.5 - 12.7)
$PaCO_2$ (kPa)	4.9 (4.4 - 5.4)	4.9 (4.4 - 5.4)	5.0 (4.7 - 5.4)
Hemoglobin, (mM)	6.4 (5.6 - 8.2)	6.6 (5.7 - 8.3)	6.2 (5.4 - 6.9)
Frequency analysis			
Mean PSD – oxyHb (μM^2/Hz) ǁ			
VLF (0.02 – 0.07 Hz)	0.31 (0.11 - 0.56)	0.34 (0.16 - 0.55)	0.20 (0.07 - 0.63)
LF (0.07 – 0.2 Hz)	0.022 (0.008 - 0.058)	0.03 (0.01 - 0.076)	0.012 (0.005 - 0.027)
Mean PSD – deoxyHb (μM^2/Hz) ǁ			
VLF (0.02 – 0.07 Hz)	0.035 (0.016 - 0.07)	0.035 (0.016 - 0.069)	0.036 (0.15 - 0.08)
LF (0.07 – 0.2 Hz)	0.003 (0.002 - 0.008)	0.003 (0.002 - 0.009)	0.002 (0.001 - 0.005)
Mean PSD ABP ($mmHg^2$/Hz) ǁ			
VLF (0.02 – 0.07 Hz)	18 (7.1 - 46)	22 (7.9 - 55)	15 (5.6 - 30)
LF (0.07 – 0.2 Hz)	2.5 (0.7 - 9.4)	3.9 (1.0 - 14)	1.6 (0.4 - 3.6)
Data overview			
Duration bedside recording (min)	125 (79 - 150)	123 (75 - 151)	132 (114 - 212)
Artefact free NIRS recording (min)	117 (66 -136)	115 (60 - 135)	125 (87 - 175)
NIRS data removed‡ (%)	8.8 (1.6 - 19)	8.2 (1.4 - 17)	9 (2.3 - 30)

* One patient can participate in both groups.
† The number of missing MAP values is for the included category n = 42 segments and for the excluded category n = 19 segments.
§ The number of missing $EtCO_2$ values are for the included category n = 92 segments and for the excluded category n = 29 segments.
ǁ The number of missing oxyHb and deoxyHb values is for both in- and excluded 4 segments as for the frequency analysis, only data with ABP, oxyHb and deoxyHb signals available were included.
‡ The removed NIRS data (before data processing) as percentage of the recorded data.
ABP = arterial blood pressure; deoxyHb = deoxyhemoglobin; LF = low frequency; oxyHb = oxyhemoglobin; PSD = power spectral density; q1 - q3 = interquartile range; VLF = very low frequency.

Table S6. Power spectral density results dichotomized for six months mortality. In this table the results of the unilateral hemispheric measurement are reported, i.e. of the hemisphere with the worst cerebral autoregulation estimate (lowest LF-phase shift for an individual).

**Median (q1-q3)**	**Total (*n*= 54)**	**Survivors (*n*= 29)**	**Non-survivors (*n*= 25)***
Mean PSD – oxyHb (*μ*M²/Hz)			
VLF (0.02 – 0.07 Hz)	0.39 (0.16 - 0.74)	0.44 (0.16 - 0.88)	0.34 (0.13 - 0.48)
LF (0.07 – 0.2 Hz)	0.02 (0.009 - 0.05)	0.02 (0.008 - 0.05)	0.03 (0.01 - 0.05)
Mean PSD – deoxyHb (*μ*M²/Hz)			
VLF (0.02 – 0.07 Hz)	0.04 (0.02 - 0.06)	0.05 (0.02 - 0.06)	0.03 (0.01 - 0.06)
LF (0.07 – 0.2 Hz)	0.003 (0.001 - 0.006)	0.003 (0.001 - 0.004)	0.004 (0.002 - 0.008)
Mean PSD ABP (mmHg²/Hz)			
VLF (0.02 – 0.07 Hz)	19 (8.1 - 45)	19 (12 - 45)	12 (4 - 44)
LF (0.07 – 0.2 Hz)	2.2 (0.9 - 6.5)	2.9 (1.1 - 5.6)	1.8 (0.26 - 9.4)

*ABP data of two patients was missing. Therefore, the two patients were excluded for the PSD analysis, as the PSD batch analysis was only done in patients with all signals available (ABP, oxyHb, and deoxyHb.
ABP = arterial blood pressure; deoxyHb = deoxyhemoglobin; LF = low frequency range; oxyHb = oxyhemoglobin; PSD = power spectral density; q1-q3 = interquartile range; VLF = very low frequency range.

Table S7. Cerebral autoregulation parameters (TFA estimates) results dichotomized for six-month mortality. In this table the results of the unilateral hemispheric measurement are reported, i.e. of the hemisphere with the worst cerebral autoregulation estimate (lowest LF-phase shift for an individual).

**Median (q1-q3)**	**Total (*n*= 54)**	**Survivors (*n*= 29)**	**Non-survivors (*n*= 25)**
Coherence			
VLF (0.02 – 0.07 Hz)	0.55 (0.43 - 0.68)	0.53 (0.47 - 0.65)	0.57 (0.41 - 0.78)
LF (0.07 – 0.2 Hz)	0.7 (0.51 - 0.86)	0.62 (0.45 - 0.76)	0.77 (0.65 - 0.87)
HF (0.2 – 0.5 Hz)	0.72 (0.57 - 0.86)	0.74 (0.57 - 0.81)	0.7 (0.59 - 0.87)
Gain			
VLF (0.02 – 0.07 Hz)	0.26 (0.17 - 0.34)	0.24 (0.17 - 0.34)	0.28 (0.19 - 0.34)
LF (0.07 – 0.2 Hz)	0.28 (0.24 - 0.34)	0.24 (0.24 - 0.38)	0.28 (0.24 - 0.32)
Phase shift (°)			
VLF (0.02 – 0.07 Hz)	39 (10 - 100)	49 (9 - 115)	35 (10 - 64)
LF (0.07 – 0.2 Hz)	12 (2.2 - 34)	20 (6.7 - 34)	6.2 (0.51 - 27)
Phase shift (°) - SD			
VLF (0.02 – 0.07 Hz)*	14 (7 - 22)	14 (5.4 - 19)	13 (7.7 - 25)
LF (0.07 – 0.2 Hz)†	6.5 (3.5 - 14)	8.3 (4 -15)	5.9 (2.9 - 9.3)
TT‡ (sec)	1.3 (0.89 - 1.6)	1.3 (1.1 - 1.1)	1.0 (0.83 - 1.3)
%BF (%)	8.8 (2.8 - 27)	9 (0.8 - 27)	8.5 (3.3 - 23)

* The number of missing SD-VLF-phase shift values: survivors *n*= 7 patients, non-survivors *n*= 3 patients.
† The number of missing SD-LF-phase shift values: survivors *n*= 6 patients, non-survivors *n*= 3 patients.
‡ The number of TT could not be defined because of predominantly blood volume oscillations. Survivors *n*= 5 patients, non-survivors *n*= 2 patients.
BF = percentage blood flow oscillations; HF = high frequency; q1 - q3 = interquartile range; LF = low frequency; TT = microvascular transit time; VLF = very low frequency.

Table S8. Patients characteristics dichotomized for six-month mortality for patients with primary neurological diagnosis

**Median (q1-q3)**	**Total (*n* = 34)**	**Survivors (*n* = 19)**	**Non-survivors (*n* = 15)**
Age (years)	56 (41 - 72)	43 (40 - 56)	72 (60 - 77)
Sex, male, *n* (%)	28 (82)	13 (68)	15 (100)
Admission diagnosis, *n* (%)			
Traumatic brain injury	13 (38)	10 (53)	3 (20)
Cardiac arrest	10 (29)	6 (32)	4 (27)
Acute stroke	9 (26)	2 (11)	7 (47)
Meningitis	1 (2.9)	1 (5.2)	0
Status epilepticus	1 (2.9)	0	1 (6.7)
APACHE IV	93 (42 - 115)	56 (22 - 97)	110 (92 - 129)
SOFA (day of measurement)	9 (7 - 10)	9 (7 - 10)	9 (7 - 11)
Length of ICU stay (days)	11 (5.3 - 19)	11 (5.5 - 21)	8.1 (4.3 - 15)
Days on mechanical ventilation	6.4 (2.1 - 12)	10 (2.5 - 13)	5.6 (2.1 - 10)
Outcome			
Mortality at ICU discharge, *n* (%)	13 (38)	-	11 (74)
GCS at ICU discharge, n (%)*			
GCS score 4-5	1 (3)	0	1 (6.7)
GCS score 6-8	1 (3)	0	1 (6.7)
GCS score 9-12	5 (15)	5 (26)	0
GCS score 13-15	13 (38)	13 (68)	0
GOSE at 6-month, *n* (%)			
Favorabel outcome, GOSE (5 - 8)	15 (44)	15 (79)	-
Unfavorable outcome, GOSE (2- 4)	4 (12)	4 (21)	-
Mortality, GOSE 1	15 (44)	19 (0)	15 (100)

*The number of missing GCS values for survivors *n* = 1.
APACHE IV = acute physiology and chronic health evaluation; GCS = Glasgow coma scale; GOSE = Glasgow outcome score extended; ICU = intensive care unit; SOFA = sequential organ failure assessment; q1 - q3 = interquartile range.

Table S9. NIRS data length and quality dichotomized for six-month mortality in patients with primary neurological diagnosis. In this table the results of the unilateral hemispheric measurement period are reported, i.e. of the hemisphere with the worst cerebral autoregulation estimate (lowest LF-phase shift for an individual).

**Median (q1 - q3)**	**Total (*n* = 34)**	**Survivors (*n* = 19)**	**Non-survivors (*n* = 15)**
Bilateral measurements, *n* (%)	23 (68)	11 (58)	12 (80)
Start measurement after ICU admission (h)	24 (14 - 62)	40 (16 - 71)	22 (11 - 50)
Duration bedside recording (min)	102 (61 - 140)	94 (60 - 144)	115 (73 - 132)
Artefact free NIRS recording (min)*	78 (48 - 124)	68 (46 - 114)	89 (52 - 126)
NIRS data removed† (%)	13 (5.6 - 28)	11 (6.4 - 28)	16 (5.1 - 28)
Number of segments per patient	4 (2 - 9)	4 (2 - 8)	6 (3 - 8)

*Discrepancy between artifact free NIRS recordings and number of ten-minute TFA segments is due to the requirement of ten contiguous minutes to be selected as a data segment.

†The removed NIRS data (before data processing) as percentage of the recorded data.

ICU = intensive care unit; LF = low frequency; NIRS = near infrared spectroscopy; q1 - q3 = interquartile range.

Table S10. Frequency analysis dichotomized for six-month mortality for patients with primary neurological diagnosis. The results of the unilateral hemispheric measurement are reported, i.e. of the hemisphere with the worst cerebral autoregulation estimate (lowest LF-phase shift for an individual).

**Median (q1 - q3)**	**Total (*n* = 34)**	**Survivors (*n* = 19)**	**Non-survivors (*n* = 15)**
Mean PSD - oxyHb ($\mu M^2/Hz$)			
VLF (0.02 – 0.07 Hz)	0.42 (0.15 - 0.89)	0.51 (0.15 - 0.91)	0.35 (0.13 - 0.46)
LF (0.07 – 0.2 Hz)	0.02 (0.012 - 0.05)	0.02 (0.008 - 0.05)	0.04 (0.02 - 0.05)
Mean PSD - deoxyHb ($\mu M^2/Hz$)			
VLF (0.02 – 0.07 Hz)	0.04 (0.02 - 0.06)	0.04 (0.02 - 0.06)	0.03 (0.009 - 0.05)
LF (0.07 – 0.2 Hz)	0.003 (0.001 - 0.005)	0.003 (0.001 - 0.004)	0.004 (0.002 - 0.006)
Mean PSD - ABP ($mmHg^2/Hz$)			
VLF (0.02 – 0.07 Hz)	32 (13 - 80)	31 (16 - 54)	38 (9.9 - 89)
LF (0.07 – 0.2 Hz)	3.6 (1.5 - 8.0)	3.3 (1.5 - 5.6)	4.9 (1.5 - 9.4)
ABP = arterial blood pressure; deoxyHb = deoxyhemoglobin; LF = low frequency; oxyHb = oxyhemoglobin; PSD = power spectral density; VLF = very low frequency; q1 - q3 = interquartile range.			

Table S11. Cerebral autoregulation parameters dichotomized for six-month mortality for patients with primary neurological diagnosis. The results of the unilateral hemispheric measurement are reported, i.e. of the hemisphere with the worst cerebral autoregulation estimate (lowest LF-phase shift for an individual).

**Median (q1 - q3)**	**Total (*n* = 34)**	**Survivors (*n* = 19)**	**Non-survivors (*n* = 15)**
Coherence			
VLF (0.02 - 0.07 Hz)	0.6 (0.45 - 0.79)	0.61 (0.49 - 0.73)	0.59 (0.40 - 0.82)
LF (0.07 - 0.2 Hz)	0.76 (0.52 - 0.88)	0.68 (0.44 - 0.83)	0.85 (0.75 - 0.90)
HF (0.2 - 0.5 Hz)	0.75 (0.63 - 0.88)	0.73 (0.57 - 0.84)	0.80 (0.70 - 0.92)
Gain			
VLF (0.02 - 0.07 Hz)	0.24 (0.17 - 0.30)	0.24 (0.17 - 0.34)	0.24 (0.19 - 0.29)
LF (0.07 - 0.2 Hz)	0.27 (0.24 - 0.33)	0.27 (0.24 - 0.33)	0.27 (0.24 - 0.31)
Phase shift (°)			
VLF (0.02 – 0.07 Hz)	34 (6 - 76)	49 (7.7 - 122)	33 (6 - 57)
LF (0.07 – 0.2 Hz)	10 (0.59 - 27)	13 (6.3 - 35)	0.83 (-2.8 - 13)
Phase shift – SD (°)			
VLF* (0.02 – 0.07 Hz)	8.4 (5.5 - 17)	7.2 (5.1 - 14)	13 (7.2 - 24)
LF† (0.07 – 0.2 Hz)	5.6 (3.2 - 13)	6.5 (3.8 - 14)	3.8 (2.9 - 7.4)
TT‡ (s)	1.3 (0.86 - 1.6)	1.4 (1.1 - 2.0)	0.95 (0.82 - 1.3)
%BF (%)	5.7 (1.5 - 22)	5.1 (0.7 - 25)	6.2 (2.9 - 15)

* The number of missing SD-VLF phase values (only one data segment was available) is for the survivors *n* = 5 and for non-survivors *n* = 3.
† The number of missing SD-LF phase values for survivors *n* = 4 and for non-survivors *n* = 3.
‡ The TT could not be defined because of predominantly blood volume oscillations for survivors *n* = 3 and for non-survivors *n* = 1.
%BF = percentage blood flow oscillations; HF = high frequency; LF = low frequency; SD = standard deviation; TT = microvascular transit time; VLF = very low frequency; q1 - q3 = interquartile range.

Table S12. Physiological variables during the bedside measurement dichotomized for six-month mortality for patients with primary neurological diagnosis. The results of the unilateral hemispheric measurement period are reported, i.e. of the hemisphere with the worst cerebral autoregulation estimate (lowest LF-phase shift for an individual).

**Median (q1 - q3)**	**Total (*n* = 34)**	**Survivors (*n* = 19)**	**Non-survivors (*n* = 15)**
MAP (mmHg) – segments	79 (69 - 88)	77 (72 - 85)	83 (68 - 90)
Heart rate (min^{-1})	73 (61 – 87)	67 (58 - 81)	82 (72 – 97)
$EtCO_2$ (kPa)‡	4 (3.7 - 4.7)	4.3 (3.9 - 4.8)	3.8 (3.4 – 4.7)
SpO_2 (%) §	97 (96 - 98)	97 (96 - 99)	97 (96 - 98)
Body temperature (°C) ǁ	36.8 (36.3 - 37.2)	37.1 (36.5 - 37.9)	36.7 (35.9 -36.9)
FiO_2 (%)	32 (25 - 40)	30 (25 - 37)	40 (28 - 52)
During measurement			
PaO_2 (kPa)	12.1 (10.6 - 13.3)	12.0 (10.4 - 13.3)	12.1 (11.1 - 13.8)
$PaCO_2$ (kPa)	4.9 (4.4 - 5.4)	4.9 (4.6 - 5.4)	4.9 (4.3 - 5.8)
PaO_2 / FiO_2 ratio (mmHg/%)	251 (219 - 360)	255 (236 - 360)	225 (150 - 345)
Hemoglobin (mM)	7.4 (6.3 – 8.3)	7.3 (6.3 – 8.6)	7.5 (6.2 – 8.2)

‡ The number of missing $EtCO_2$ values are for survivors *n* = 1 and for non-survivors *n* = 1.
§ The number of missing SpO_2 values is for survivors *n* = 1.
ǁ The number of missing body temperature values is for survivors *n* = 7 and for non-survivors *n* = 2.
$EtCO_2$ = end tidal carbon dioxide; FiO_2 = oxygen fraction; MAP = mean arterial blood pressure; $PaCO_2$= partial carbon dioxide pressure; PaO_2 = partial oxygen pressure; q1 - q3 = interquartile range; SpO_2 = peripheral oxygen saturation.

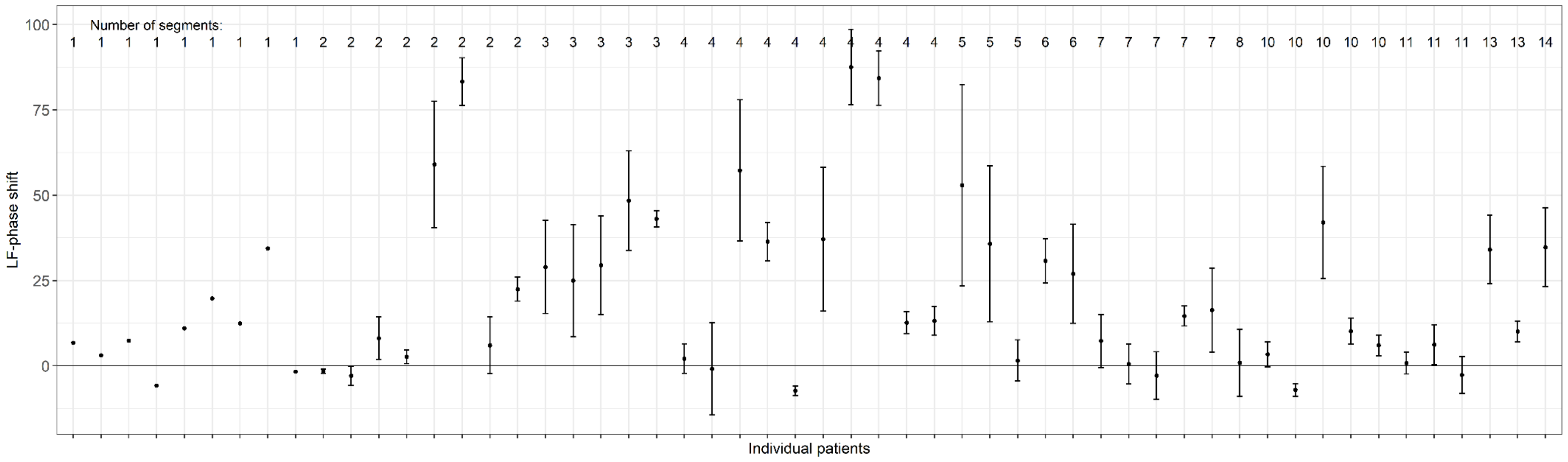

Figure S4. Within patient variability for the low frequency phase shift (*n* = 54). For each patient (x-axis) the LF-phase shift and SD of the worst hemisphere (lowest LF-phase shift for an individual) (y-axis) is shown. The number of included segments is shown on top. From left to right the number of segments per patient increases, ranging from 1 until 14 segments. The within patient variability seems independent from the number of segments. Nine patients were presented by one segment and therefore no SD was available. The median SD for *n* = 45 patients was 6.5^{o} (3.5 - 14) (Supplementary Table S7). deoxyHb = deoxyhemoglobin; LF = low frequency ; oxyHb = oxyhemoglobin ; SD = standard deviation.

Table S13. Multivariate logistic regression model for patients with primary neurological diagnosis (*n* = 34)

**Independent variable**	**Adjusted OR (95% CI)**	***p*-value**
LF-phase shift (/10°)	0.27 (0.10 - 0.78)	0.015
Age (years)	1.16 (1.01 - 1.33)	0.032
APACHE IV	1.00 (0.97 - 1.04)	0.614
Measurement time after ICU admission (h)	1.51 (0.78 - 2.90)	0.218

APACHE IV = acute physiology and chronic health evaluation IV ; CI = confidence interval ; ICU= intensive care unit; LF = low frequency; OR = odds ratio.

REFERENCES

1. Duncan, A.; Meek, J.H.; Clemence, M.; Elwell, C.E.; Fallon, P.; Tyszczuk, L.; Cope, M.; Delpy, D.T. Measurement of Cranial Optical Path Length as a Function of Age Using Phase Resolved near Infrared Spectroscopy. *Pediatr. Res.* **1996**, *39*, 889–894, doi:10.1203/00006450-199605000-00025.
2. Elting, J.W.; Sanders, M.L.; Panerai, R.B.; Aries, M.; Bor-Seng-Shu, E.; Caicedo, A.; Chacon, M.; Gommer, E.D.; Van Huffel, S.; Jara, J.L.; et al. Assessment of Dynamic Cerebral Autoregulation in Humans: Is Reproducibility Dependent on Blood Pressure Variability? *PLoS One* **2020**, *15*, e0227651, doi:10.1371/journal.pone.0227651.
3. Claassen, J.A.H.R.; Meel-van den Abeelen, A.S.S.; Simpson, D.M.; Panerai, R.B. Transfer Function Analysis of Dynamic Cerebral Autoregulation: A White Paper from the International Cerebral Autoregulation Research Network. *J. Cereb. blood flow Metab. Off. J. Int. Soc. Cereb. Blood Flow Metab.* **2016**, *36*, 665–680, doi:10.1177/0271678X15626425.
4. van Beek, A.H.; Claassen, J.A.; Rikkert, M.G.O.; Jansen, R.W. Cerebral Autoregulation: An Overview of Current Concepts and Methodology with Special Focus on the Elderly. *J. Cereb. blood flow Metab. Off. J. Int. Soc. Cereb. Blood Flow Metab.* **2008**, *28*, 1071–1085, doi:10.1038/jcbfm.2008.13.
5. von Elm, E.; Altman, D.G.; Egger, M.; Pocock, S.J.; Gøtzsche, P.C.; Vandenbroucke, J.P. The Strengthening the Reporting of Observational Studies in Epidemiology (STROBE) Statement: Guidelines for Reporting Observational Studies. *J. Clin. Epidemiol.* **2008**, *61*, 344–349, doi:10.1016/j.jclinepi.2007.11.008.
6. Elting, J.W.J.; Tas, J.; Aries, M.J.; Czosnyka, M.; Maurits, N.M. Dynamic Cerebral Autoregulation Estimates Derived from near Infrared Spectroscopy and Transcranial Doppler Are Similar after Correction for Transit Time and Blood Flow and Blood Volume Oscillations. *J. Cereb. blood flow Metab. Off. J. Int. Soc. Cereb. Blood Flow Metab.* **2020**, *40*, 135–149, doi:10.1177/0271678X18806107.
